# The relationship between sugar-sweetened beverages, sleep disorders, and diabesity

**DOI:** 10.3389/fendo.2022.1041977

**Published:** 2023-01-09

**Authors:** Yi Zhang, Chao Liu, Yijing Xu, Yanlei Wang, Yulin Zhang, Tian Jiang, Qiu Zhang, the China National Diabetic Chronic Complications Study Group

**Affiliations:** ^1^ Department of Endocrinology, The First Affiliated Hospital of Anhui Medical University, Hefei, Anhui, China; ^2^ Department of Maternal, Child and Adolescent Health, School of Public Health, Anhui Medical University, Hefei, Anhui, China; ^3^ The Second Clinical Medical College, Anhui Medical University, Hefei, Anhui, China

**Keywords:** diabesity, BMI, waist circumference, sugar-sweetened beverage, diabetes

## Abstract

**Background:**

Diabetes and obesity in adults are global issues. Obesity and type 2 diabetes mellitus (T2DM) are increasingly categorized under the umbrella term “diabesity.” Health risk factors (HRFs), which include altering sleep habits and reducing sugar-sweetened beverages (SSBs) consumption, have emerged as relatively novel and crucial strategies for preventing and treating diabetes.

**Objective:**

We aimed to explore: 1) whether SSBs could affect diabesity in China’s community; 2) whether HRFs could moderate this relationship; and 3) whether a three-way interaction exists between HRFs, SSBs, and diabesity.

**Methods:**

On December 10, 2018, we investigated diabetes complications in four cities in Anhui Province and obtained basic and lifestyle information using a detailed questionnaire. The primary exposure was SSBs and outcomes were body mass index (BMI) and waist circumference (WC), while glycated hemoglobin (HbA1c) and sleep patterns (including duration and disorders) were considered moderators.

**Results:**

Overall, 1920 participants were enrolled, and those who did not complete the questionnaire were excluded. Finally, this study included 1765 participants, with a response rate of 92.0%. The mean age was (57.10 ± 10.0) years. Patients with lower educational levels were more likely to have a lower prevalence of WC (*χ* ^2^ = 2.73) and BMI (*χ* ^2^ = 3.47), and some HRFs were positively correlated with WC and BMI. Additionally, SSBs were significantly associated with BMI (β = 1.29) and WC (β = 2.97), and there was also differences based on sex, some HRFs, such as HbA1c, FBG and TG, showed higher levels in male participants, whereas TC level was higher in female participants. In the moderation analysis, sleep patterns were also associated with total cholesterol, triglyceride, and BMI. Furthermore, there were three-way interaction effects among HbA1c, sleep patterns, and SSBs on total cholesterol, triglyceride, BMI, and WC. Moreover, sensitivity analysis demonstrated that our results were robust.

**Conclusion:**

SSBs positively correlated with patterns dose-dependently. Moreover, SSBs could also be associated with sleep patterns, and blood glucose levels were correlated with diabesity. A three-way interaction effect was discovered between SSBs, sleep patterns, blood glucose levels, and patterns. Therefore, understanding the diabesity caused by SSBs and other HRFs can help prevent its occurrence.

## Introduction

As a common metabolic disease, diabetes is one of the leading causes of death and disability worldwide, with about one in ten adults reported to have type 2 diabetes mellitus (T2DM) (1, 2). In 2021, the International Diabetes Federation (IDF) predicted that the population of individuals with diabetes would increase to 597 million (3). Individuals with T2DM also have a significantly increased risk of adverse outcomes such as cardiovascular disease (CVD), and cardiometabolic syndrome is connected to an increased risk of diabetes (4). Additionally, diabetic dyslipidemia and obesity have emerged as key biomarkers for an increased CVD risk in patients with diabetes. Therefore, early detection and active treatment of dyslipidemia and obesity prevention are crucial to save the lives of patients with diabetes and preventive the onset of atherosclerotic CVD. Among them, diabesity is a terminology that refers to the combined negative health impacts of obesity and T2DM (5), and the global obesity and T2DM epidemic is a critical public health concern (6). Therefore, “diabesity” is more often used to describe the harmful effects of obesity and diabetes on health. Furthermore, high body mass index (BMI) is independently associated with an elevated coronary plaque burden in patients with diabetes, providing indirect evidence for the concept of diabesity as a dangerous mixture of obesity and T2DM (7).

Sugar-sweetened beverage (SSB) intake is strongly associated with obesity, and weight gain and obesity have also long been strongly associated with CVD. According to epidemiological studies, SSB intake is a key component of dietary sugar intake and is associated with metabolic syndrome, hypertension and obesity (8). Additionally, many estimated deaths were caused by diabetes (72.3%), followed by CVD (24.2%) and BMI-related cancers (3.5%), according to country-level data (9). In 2010, 8 526 456 disability-adjusted life years (DALYs) were attributed to SSBs, with approximately 75% of deaths prevalent in low-and middle-income countries (9). Furthermore, several meta-analysis also have suggested the relationship between SSBs intake and diabetes, obesity, and CVD (10, 11).

Previous articles have reviewed the temporal patterns of SSB intake and the corresponding clinical impacts on the risk of obesity, T2DM, and CVD (10, 11). Furthermore, individuals with diabetes also have a pattern of increased triglycerides (TG), small dense low-density lipoprotein cholesterol (LDL), and low levels of high-density lipoprotein (HDL) cholesterol, which is known as diabetic dyslipidemia (12). They also highlighted the underlying biological processes, clinical implications, and methodological difficulties inherent in the literature, the latter including the interaction of these factors, which has not been reported accordingly. Therefore, gaining insights into the effects of SSBs on dyslipidemia and diabetes control is of utmost importance.

Although most studies have focused on the negative effects of SSBs, other factors seem involved, and these risk factors may have previously interacted. The cause of diabetes combined with obesity is usually abnormal rhythm and other reasons. Interestingly, researchers have embraced sleep disorders, which contribute to dysregulated metabolic physiology, as novel risk factors associated with metabolism. For example, Ogilvie and Patel reported a relationship between T2DM risk and sleep-related exposures–problems such as poor sleep habits and sleep disturbances are very common in adults with T2DM (13). Furthermore, Poggiogalle et al. demonstrated that the normal circadian rhythm system regulates the normal metabolic process of the body by controlling the changes of glucose and lipid homeostasis and energy metabolism. The disruption of these rhythms will impair metabolism and become the etiology of metabolic diseases. In addition, the authors concluded that circadian disruption caused by poor sleep or unhealthy eating habits may impair metabolic health and increase the development of disease. Thus, these associations between the circadian system, metabolism, and behavior highlight the relevance of chronobiology in the prevention and treatment of type 2 diabetes, obesity, and hyperlipidemia (14).

Generally, the evidence showing that reducing SSBs lowers the risk of obesity and associated diseases such as T2DM is fascinating, and the correlation between SSBs and diabetes has received special attention. Currently, T2DM has been shown to be preventable. While weight control is a major factor, physical activity also has an impact, and dietary factors such as reducing saturated fat, increasing intake of fruits and vegetables, dietary fiber, and sleeping habits can have an effective impact on diabetes management (14, 15). Furthermore, these studies on health risk factors (HRFs), such as SSBs and sleep disorders, can promote the formation and development of diabetes management approaches and provide indirect evidence. Similarly, HRFs and establishing HRFs-diabesity-focused research requires a comprehensive model. Consequently, the phenomenon of middle-aged and older people with diabetes and obesity co-occurrence and clustering of diabetes should be considered comprehensively, involving a combination of behavior, diet, and sleep rhythms to achieve desired results.

**Figure 1:**
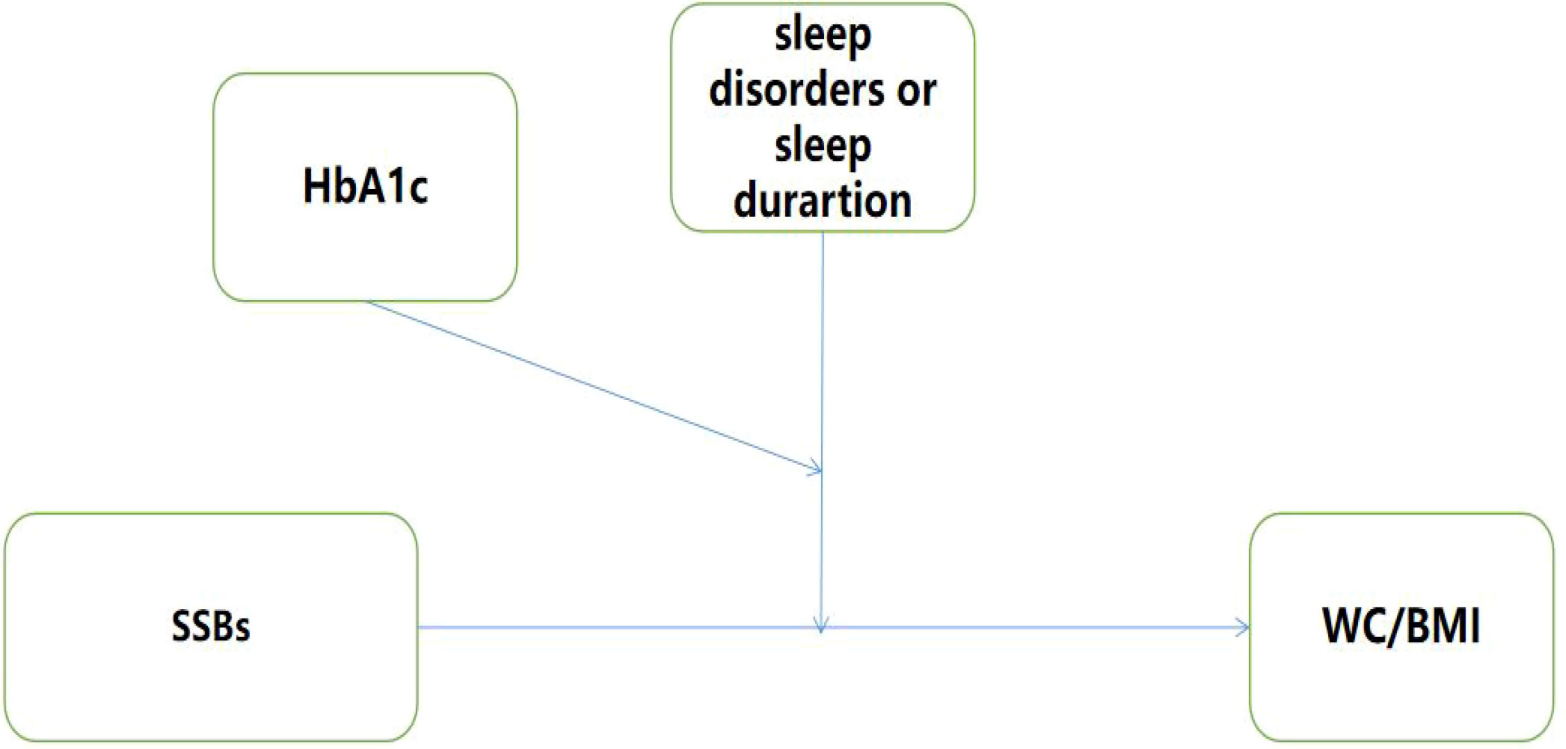
Hypothesis model.

However, to our knowledge, there is no studies to date have investigated whether measured SSB consumption is associated with sleep deficiency and sleep disorders in patients with diabesity from different regions. Understanding the association between sleep patterns and SSBs consumption in participants with diabetes and obesity and observing these relationships through glucose monitoring is essential to inform public health policiers and tailor interventions. Therefore, this study aimed to explore the correlation between metabolic indices and diabetes and to explore whether metabolic indices combined with HRFs were associated with diabetes. We hypothesized the following: 1) SSBs could affect diabesity in China’s community; 2) HRFs could moderate this relationship; and 3) there is a three-way interaction between HRFs, SSBs, and diabesity (**Figure 1**).

## Materials and methods

### Survey subjects

This cross-sectional study aimed to explore the association between HRFs and diabetes and its complications. This study was designed and reported following the Strengthening the Reporting of Observational Studies in Epidemiology (STROBE) checklist.

### Study design

In 2018, the Diabetes Society of the Chinese Medical Association, China Center for Chronic Noncommunicable Disease Control and Prevention, Chinese Center for Disease Control and Prevention, and Bethune Foundation screened for chronic complications of diabetes in 31 provinces/autonomous regions/municipalities across China (16). The survey was conducted based on the China Chronic Disease and its Risk Factors Monitoring System, which covers 31 provinces (autonomous regions and municipalities directly under the central government). Furthermore, multi-stage stratified random sampling was employed for the respondent selection. Detailed sampling techniques are provided in the **Supplementary Method**.

### Sample size estimation

Based on previous studies, diabetes prevalence was 11.2%, corresponding to the trends in obesity and overweight, with relative precision of 15% (ε), α = 0.05, Z_1-α/2_ = 1.96. Therefore, applying the following formula, the minimum sample size was determined to be 690. Furthermore, given a multi-center design with different ages and regions, analysis, and future follow-up needs, this minimum requirement was used for grade sampling in all regions to ensure that the analysis was performed at multiple stratification levels in each community. Finally, 1400 participants were surveyed.


$$\text{n}=\frac{\left(1-\text{p}\right){\text{ Z}}_{1-\text{α}/2}}{{�}^{2}p}$$

### Inclusion criteria

1) obtained informed consent from the patients, 2) were 18 years of age and older and had lived in the survey area for at least 6 months during the 12 months before the survey, and 3) were diagnosed with diabetes.

### Exclusion criteria

**Figure 2:**
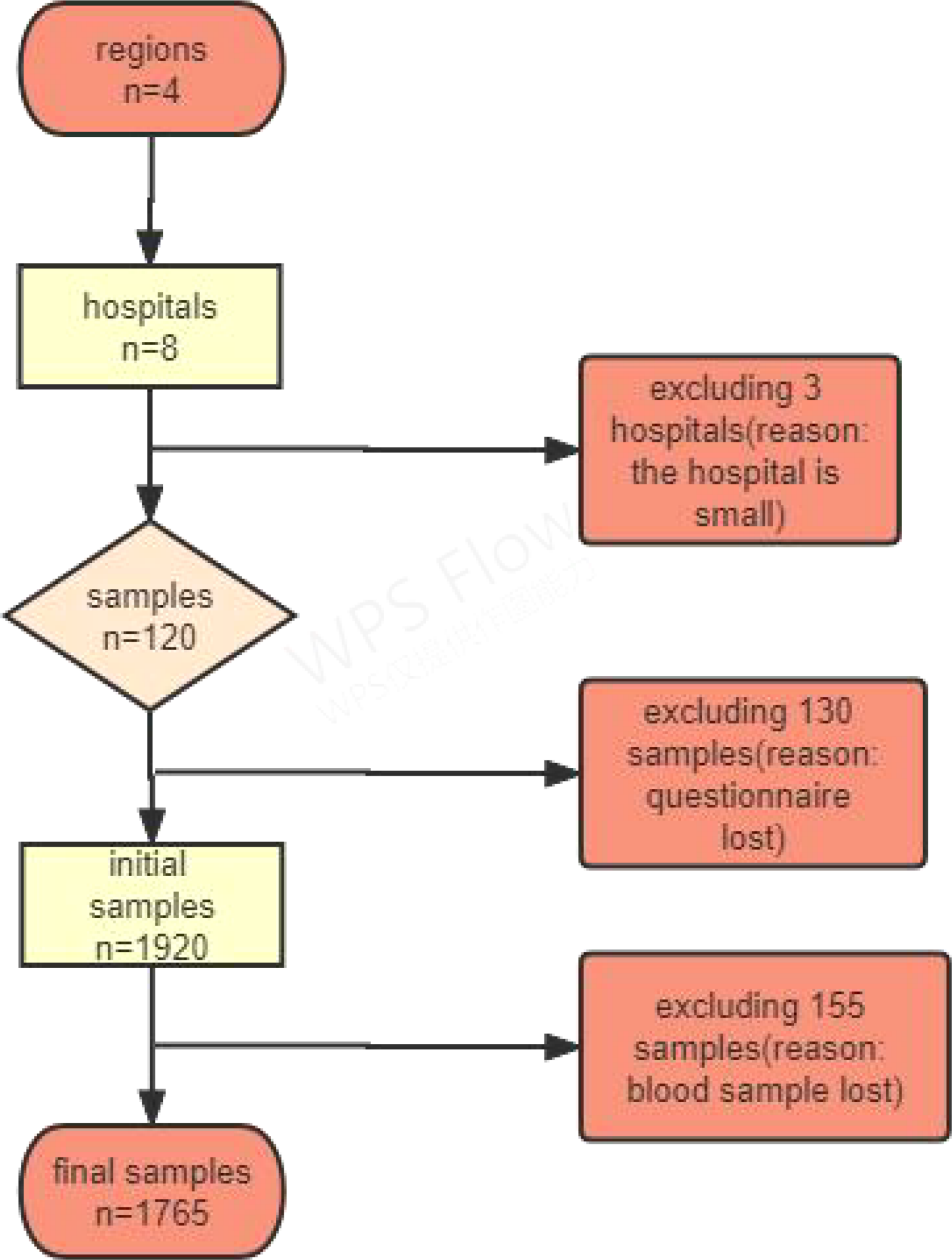
Flow chart of participants in study.

1) informed consent was not obtained; 2) pregnant women and those with mental illness were excluded; 3) failure to submit a questionnaire; 4) congenital or acquired immunodeficiency; and 5) incomplete medical examination. **Figure 2** shows the study’s flowchart.

### Investigation contents and methods

#### Questionnaire survey

The personal questionnaire was administered by a uniformly trained investigator using a tablet computer in the form of a face-to-face interview since the respondents could not fill it out. The questionnaire included sociodemographic characteristics and HRFs such as smoking, drinking alcohol, dietary behaviors, physical activity, screen time, SSB intake, sleep disorders, sleep duration, and sedentary behavior. When the mentioned questions were difficult to answer, they were answered by a family member most familiar with the situation.

SSBs were measured as follows: “Please recall whether you regularly consumed any of the following foods in the previous 12 months, and estimate the frequency and amount consumed.” Participants answered, “Whether or not to eat, the frequency of consumption, and the amount of each consumption.” The consumption of each SSB was employed in the study’s analysis (16). Furthermore, sleep disorders were assessed as follows: Have you had the following sleep complications at least 3 days weekly in the past 30 days?” including snoring, choking, or suffocating; difficulty falling asleep (more than 30 minutes); more awakening twice (including twice); waking up early, and difficulty in sleeping again; have you taken sleeping pills (Western medicine or traditional Chinese medicine) for at least 1 day in the past 30 days to help you sleep. The participants answered yes or no. Subsequently, we summed these answers into a total score and further manipulated other analyses. Sleep duration was measured as follows:

#### Body measurement

Body measurements included height, weight, WC, blood pressure, and heart rate (HR). Height was measured as tide gauge zero (TGZ) height with a maximum scale of 2.0 m and precision of 0.1 cm; weight was measured using a Billiida (TANITA) HD-390 with a minimum unit of 0.1 kg; WC was measured with a maximum scale of 1.5 m, 1 cm width, and precision of 0.1 cm; blood pressure and HR measurements using the Omron HBP1300 electronic sphygmomanometer.

#### Laboratory testing

Each subject’s venous blood sample (5 ml) was extracted and separated into fluoxalic acid, ethylenediaminetetraacetic acid (EDTA), and standard test tubes. All direct biochemical measurements were performed using automated chemical analyzers and off-the-shelf reagent kits following the manufacturer’s standardized protocols. Venous blood was collected 10–12 h later, and random urine samples were collected. Detection measures included fasting plasma glucose, glycated hemoglobin (HbA1c), blood lipids (serum TC and serum TG, among others), and blood glucose was tested by the local laboratory that passed the survey site. Next, other blood and urine samples were centrifuged and packaged at the investigation site and preserved as required before being collected, frozen, and transported to the National Project Working Group-designated medical inspection institution for testing and preservation.

### Covariates

Sex, age, educational level, residential area, overall annual household income, marital status, and ethnicity were included as covariates.

### Sensitivity analysis

Here, sensitivity analysis was used to test the robustness of the model: (1) Model 1 did not control for covariates, and Model 2 controlled for covariates. (2) We also explored the relationship between HbA1c, SSBs, sleep disorders, and TC and TG **(Supplement Results)**. (3) We also examined the association between different HRFs, such as physical activity (moderate), sedentary behavior, walking, alcohol drinking, smoking, sleep duration, BMI, and WC **(Supplement Results,** **Tables S5**–**S12** **)**.

### Statistics analysis

Statistical analysis was performed using the Statistical Package for Social Sciences (SPSS) version 23.0. Continuous and categorical variables were described as mean ± standard deviations (SD) using one-way analysis of variance (ANOVA) and as frequency and percentage using the chi-square test, respectively. Additionally, multivariate logistic regression was used to analyze the correlation between HRFs and BMI, WC, and other metabolic indices. Finally, the missing data were processed using SPSS version 23.0 after preliminary data sorting. Generally, the missing data rate in this study was minimal, and that for each item was< 1%. Therefore, a multiple imputation method is adopted in the SPSS version 23.0 to analyze the missing data at the project level. The main steps are as follows.

Step 1 entailed descriptive statistics about the correlation between sociodemographic factors, HRFs, BMI, and WC. In step 2, multilevel logistic regression was used to test the relationship between HRFs, BMI, and WC. The PROCESS moderation program was employed to perform the moderation analysis in Step 3 (17) in assessing the moderating effect between HRFs, BMI, and WC. In Step 4, we tested another moderating effect between HRFs, TG, and TC. Additionally, in the SPSS PROCESS, the interacting effect is calculated automatically through the software. It produces the proportion of variance explained by the moderating effect of BMI (R^2^ increases due to the interaction). Finally, we adjusted for the impacts of sociodemographic correlates in the moderation model. The moderating effect was considered significant when the 95% confidence interval (CI) did not contain zero. This study was approved by the Ethics Committee of the First Affiliated Hospital of Anhui Medical University (Ethics Approval No: PJ2019-09-05), and all participants provided written informed consent.

## Results

### The prevalence characteristics of waist circumference

Overall, 1920 participants were included, of which those who did not complete the questionnaire were excluded, leaving 1765 participants. The mean age was 59.10 ± 10.0 years, and 93.6% of the participants did not consume SSBs. Additionally, BMI prevalence was 34.8% (obese), 36.7% (overweight), 1.0% (underweight), and 27.5% (normal weight). Furthermore, the results of the relationship between general sociodemographic indicators and WC showed that a higher educational level was more likely to have a reduced WC prevalence; moreover, sex, marital status, ethnicity, age, and total annual household income (Yuan) were also not correlated with WC (**Table 1**).

**Table 1:** The prevalence characteristics of waist circumference and BMI.

Variables	Waist circumference (cm)				BMI (kg/m^2^)			
	N (%)	Average	Standard deviation	*t/F* value	N (%)	Average	Standard deviation	*t/F* value
sex				5.87				0.50^*^
Male	874	91.09	9.66		874	25.97	3.59	
Female	891	88.35	9.93		891	26.07	3.72	
Married status				-0.40				-1.13
Unmarried	30	88.99	12.38		30	25.27	3.90	
Married	1735	89.72	9.84		1735	26.04	3.65	
Ethnic				0.10				0.22
Han	1745	89.71	9.86		1745	26.02	3.65	
Non-Han	20	89.49	12.63		20	25.84	4.15	
Educational level				2.73^*^				3.47^*^
Under primary	800	89.38	9.58		800	26.16	3.61	
Primary	262	89.70	9.67		262	25.79	3.55	
Junior high	472	90.74	10.24		472	26.23	3.90	
Senior high above	231	88.75	10.32		231	25.40	3.34	
Total annual household income (yuan)				1.90				1.19
≤18000	354	89.53	10.63		354	26.0	4.14	
18000-40000	749	90.31	9.39		749	26.17	3.48	
40000-70000	346	89.37	9.69		346	26.02	3.41	
>70000	316	88.85	10.33		316	25.71	3.75	
Age (years)				0.078				5.96^*^
≤43	194	89.73	11.93		194	26.540	4.41	
44-59	823	89.66	9.60		823	26.084	3.65	
≥60	748	89.75	9.63		748	25.821	3.44	

*P < 0.05.

Conversely, the results of the general demographic indicators and BMI showed that lower educational level, female sex, and age group were more likely to have a higher BMI prevalence; moreover, married status and ethnicity, and total annual household income (Yuan) were also unassociated with BMI (**Table 1**).

### The multilevel linear regression between dependent health risk factors (HRFs) and WC

**Table 2** shows some health risk behaviors positively correlated with WC when adjusting for educational level, marital status, ethnicity, sex, and age. Furthermore, sedentary behavior, walking, sleep disorders, SSBs, and HbA1c and TG levels correlated dose-dependently. Moreover, no correlation was observed between moderate physical activity, smoking, sleep duration, and WC.

**Table 2:** The multilevel linear regression between dependent health risk factors and WC.

Variables	WC (cm)					BMI (kg/m^2^)				
	R^2^	t	F	P	β (95%CI)	R^2^	t	F	P	β (95%CI)
Physical activity (moderate)	0.02	-0.79	5.95	>0.05	-0.29 (-0.99,0.42)	0.01	0.83	2.63	>0.05	0.21 (-0.29,0.71)
Sedentary behavior	0.02	1.68	6.27	>0.05	0.15 (-0.03,0.33)	0.01	2.27	3.38	<0.05	0.42 (0.06,0.78)
Walking	0.02	-2.12	7.31	<0.05	-0.12 (-0.24,-0.01)	0.01	-2.04	3.23	<0.05	-0.004 (-0.01,-0.001)
Sleep (dichotomy)	0.02	1.72	7.10	>0.05	-0.55 (-0.12,1.76)	0.01	0.78	2.6	>0.05	0.14 (-0.21,0.49)
Alcohol drinking	0.02	1.75	7.11	>0.05	0.97 (-0.12,2.07)	0.01	-1.72	3.02	>0.05	-0.32 (-0.68,0.04)
Smoking	0.02	0.38	6.62	>0.05	0.24 (-1.01,1.49)	0.01	-0.42	2.55	>0.05	-0.19 (-1.10,0.72)
SSBs	0.03	3.11	8.24	<0.01	2.97 (1.09,4.84)	0.02	3.63	4.74	<0.01	1.29 (0.59,1.99)
Sleep disorders	0.03	4.64	9.00	<0.01	0.87 (0.50,1.24)	0.02	4.71	5.12	<0.01	0.33 (0.19,0.47)
Sleep duration	0.02	-1.03	6.72	>0.05	-0.14 (-0.40,0.13)	0.01	-0.21	2.54	>0.05	-0.01 (-0.11,0.09)
HbA1c	0.03	2.07	7.40	<0.05	0.28 (0.014,0.54)	0.01	-0.52	2.62	>0.05	-0.03 (-0.12,0.07)
FBG	0.02	0.19	6.68	>0.05	0.02 (-0.13,0.16)	0.01	-2.04	3.27	<0.05	-0.06 (-0.11,-0.002)
TG	0.04	6.23	13.33	<0.01	0.55 (0.38,0.72)	0.03	6.46	9.60	<0.01	0.21 (0.15,0.28)
TC	0.02	0.58	6.78	>0.05	0.11 (-0.27,0.50)	0.01	-0.44	2.61	>0.05	-0.03 (-0.18,0.11)

Controlled for educational level, total annual household income, marital status, ethnic, sex and age.

Additionally, after controlling for covariates, some health risk factors were positively correlated with BMI (**Table 2**). Sedentary behavior, walking, sleep disorders, SSBs, FBG, and TG were also related dose-dependently. Furthermore, no correlation existed between moderate physical activity, smoking, HbA1c levels, sleep duration, and BMI. **Table 2** shows the correlations between health risk factors, BMI, WC, and SSBs.

We stratified health risk factors by sex, and there were differences in sleep disorders (β=6.85), HbA1c (β=7.68), FBG (β=9.63), TG (β=2.48), TC (β=5.11), alcohol drinking (*χ^2 =^* 340.86), smoking (*χ^2 =^* 625.82) between sexes (**Tables S1**, **S2** **)**. Additionally, there were difference between sexes in sedentary behavior, SSBs, sleep disorders and TG (**Tables S3**, **S4** **)**.

### The moderation analysis between sex, SSBs, sleep disorders, BMI, and WC

Accordingly, moderation analyses were performed using educational level, marital status, ethnicity, and age as the control variables. **Tables 3**, **4** shows the results. Notably, the diet was significantly associated with BMI severity. Furthermore, sleep disorders were also significantly linked to BMI as well as diet × sleep disorders, which were considerably associated with BMI and WC.

**Table 3:** The moderation analysis between SSBs, Sleep disorders, HbA1c and WC.

Variables	WC					
	coeff	se	t	P	LLCI	ULCI
Sleep disorders	5.56	2.66	2.09	<0.05	0.35	10.78
SSBs	34.54	17.13	2.02	<0.05	0.93	68.14
Sex	17.25	11.82	1.46	>0.05	-5.94	40.44
Int_1	-5.16	2.44	-2.12	<0.05	-9.94	-0.39
Int_2	-22.64	10.85	-2.09	<0.05	-43.92	-1.35
Int_3	-3.32	1.65	-2.02	<0.05	-6.55	-0.09
Int_4	3.62	1.51	2.40	<0.05	0.66	6.58

Int 1: SSBs × sleep disorders; Int 2: SSBs × sex; Int 3: sex × sleep disorders; Int 4: SSBs × sleep disorders × sex.

**Table 4:** The moderation analysis between SSBs, Sleep disorders, HbA1c and BMI.

Variables	BMI					
	coeff	se	t	P	LLCI	ULCI
Sleep disorders	1.39	0.99	1.41	>0.05	-0.55	3.34
SSBs	8.66	6.40	1.35	>0.05	-3.88	21.21
Sex	5.55	4.41	1.26	>0.05	-3.10	14.21
Int_1	-1.27	0.91	-1.40	>0.05	-3.06	0.51
Int_2	-6.45	4.05	-1.59	>0.05	-14.39	1.49
Int_3	-0.95	0.61	-1.54	>0.05	-2.15	0.26
Int_4	1.06	0.56	1.88	0.06	-0.05	2.16

Int 1: SSBs × sleep disorders; Int 2: SSBs × sex; Int 3: sex × sleep disorders; Int 4: SSBs × sleep disorders × sex.

### The moderation analysis between HbA1c, SSBs, sleep disorders, BMI, and WC

Moderation analyses were performed with educational level, marital status, ethnicity, sex, and age as control variables (**Table 5**). SSBs were significantly associated with BMI severity as well as sleep disorders. Furthermore, SSBs and sleep disorders were also significantly associated with BMI.

**Table 5:** The moderation analysis between SSBs, sleep disorders and BMI.

Variables	BMI					
	coeff	se	t	P	LLCI	ULCI
Sleep disorders	-1.80	0.80	-2.25	<0.05	-3.37	-0.23
SSBs (dichotomy)	-2.50	1.26	-1.98	<0.05	-4.98	-0.03
Int_1	2.28	0.74	3.10	<0.01	0.84	3.73

Int 1: SSB × sleep disorders.

Additionally, moderation analyses were performed with educational level, total annual household income, marital status, ethnicity, sex, and age as control variables **(** **Tables 6**, **7** **)**. First, SSBs and sleep disorders were not significantly associated with BMI and WC prevalence. However, HbA1c significantly correlated with the level of BMI. Second, no significant effects were observed between sleep disorders × SSBs, SSB × HbA1c, and HbA1c × sleep disorders on BMI, respectively. Finally, a three-way interaction effect was observed between HbA1c, sleep disorders, and SSBs on BMI. There is no significant effects between HbA1c, sleep disorders, and SSBs on WC.

**Table 6:** The moderation analysis between SSBs, Sleep disorders, HbA1c and BMI.

Variables	BMI					
	coeff	se	t	P	LLCI	ULCI
Sleep disorders	3.36	2.07	1.62	>0.05	-0.71	7.42
SSBs	10.64	8.77	1.21	>0.05	-6.55	27.83
HbA1c	4.67	2.17	2.15	<0.05	0.42	8.92
Int_1	-1.54	1.07	-1.43	>0.05	-3.64	0.57
Int_2	2.03	1.12	-1.81	>0.05	-4.23	0.17
Int_3	0.59	0.26	-2.27	<0.05	-1.11	-0.082
Int_4	0.28	0.14	1.90	<0.05	0.01	0.0035

Int 1: SSBs × sleep disorders; Int 2: SSBs × HbA1c; Int 3: HbA1c × sleep disorders; Int 4: SSBs × sleep disorders × HbA1c.

**Table 7:** The moderation analysis between SSBs, Sleep disorders, HbA1c and WC.

Variables	WC					
	coeff	se	t	P	LLCI	ULCI
Sleep disorders	7.27	5.58	1.30	>0.05	-3.68	18.22
SSBs	22.27	23.59	0.94	>0.05	-23.99	68.54
HbA1c	9.90	5.83	1.70	>0.05	-1.54	21.34
Int_1	-3.28	2.89	-1.14	>0.05	-8.95	2.38
Int_2	-3.98	3.02	-1.32	>0.05	-9.90	1.94
Int_3	-1.23	0.70	-1.75	>0.05	-2.61	0.15
Int_4	0.51	0.37	1.41	>0.05	-0.20	1.23

Int 1: SSBs × sleep disorders; Int 2: SSBs × HbA1c; Int 3: HbA1c × sleep disorders;

Int 4: SSBs × sleep disorders × HbA1c.

Furthermore, similar analyses were performed with educational level, marital status, total annual household income, ethnicity, sex, and age as control variables (**Tables 8**). First, SSBs, sleep duration, and HbA1c were not significantly related to WC prevalence. Second, there were also no significant effects between sleep disorders × SSBs, SSBs × HbA1c, and HbA1c × sleep disorders on WC, respectively. Furthermore, no three-way interaction effects between HbA1c, sleep disorders, and SSBs on WC. We also examined the relationship between SSBs, sleep duration, HbA1c level, and WC. There is also significant effects between HbA1c, sleep duration, and SSBs on BMI.

**Table 8:** The moderation analysis between SSBs, Sleep duration, HbA1c and WC.

Variables	WC					
	coeff	se	t	P	LLCI	ULCI
SSBs	-0.03	0.02	-1.44	>0.05	-0.06	0.01
Sleep duration	0.43	0.21	2.07	<0.05	0.02	0.83
HbA1c	0.49	0.23	2.17	<0.05	0.05	0.94
Int_1	0.003	0.002	1.76	>0.05	-0.0004	0.01
Int_2	0.01	0.002	2.09	<0.05	0.0003	0.01
Int_3	-0.07	0.03	-2.26	<0.05	-0.12	-0.01
Int_4	-0.001	0.0003	-2.18	<0.05	-0.001	-0.0001

Int 1: SSBs × sleep duration; Int 2: SSBs × HbA1c; Int 3: HbA1c × sleep duration;

Int 4: SSBs × sleep duration × HbA1c.

### The mediated moderation analysis between HbA1c, SSBs, sleep disorders, BMI, and WC

We also performed moderated mediation analyses were shown in **Tables 9**, **10**. The results demonstrated that HbA1c levels significantly moderated the effects of HRFs on BMI and WC. Remarkably, the mediation of sleep disorders on BMI and WC was significantly moderated by the HbA1c level. Similar results were shown in **Tables 11**, **12**.

**Table 9:** Model characteristics for the conditional process analysis.

Variables	Sleep disorders			WC		
	B	t value	*P* value	B	t value	*P* value
SSBs	-0.83	-1.73	>0.05	-5.70	-1.54	>0.05
HbA1c	-0.20	-1.79	>0.05	-2.07	-1.84	>0.05
SSBs*Sleep disorders	0.10	1.70	0.08	0.35	0.76	>0.05
Sleep disorders				-1.15	-1.38	>0.05
Sleep disorders*HbA1c				0.25	2.29	<0.05
R^2^	0.002					
F	1.16					

Mediate variables: sleep disorders, moderated variables: HbA1c, independent variables: SSBs, dependent variables: WC. The model was uncontrolled covariates.

**Table 10:** Bootstrapped conditional direct and indirect effects.

			BMI		
Direct effect			Effect	SE	(LL,UL)
	Predictor	BMI			
	Moderator (HbA1c)	Low	-3.67	1.32	-6.26,-1.07
		Medium	-3.06	0.96	-4.94,-1.17
		High	-2.45	1.17	-4.75,-0.15
Indirect effect			Effect	SE	(LL,UL)
	Predictor	BMI			
	Mediator (sleep disorders)	Low	5.83	-0.07	-0.40,0.04
		Medium	7.58	-0.05	-0.25,0.13
		High	9.33	0.12	-0.16,0.54

**Table 11:** Model characteristics for the conditional process analysis.

Variables	Sleep disorders			BMI		
	B	t value	*P* value	B	t value	*P* value
SSBs	-0.83	-1.73	>0.05	-2.29	-1.67	>0.05
HbA1c	-0.20	-1.79	>0.05	-0.98	-2.37	<0.05
SSBs*Sleep disorders	0.10	1.70	0.08	0.11	0.67	>0.05
Sleep disorders				-0.98	-1.58	>0.05
HbA1c *Sleep disorders				0.11	2.69	<0.01
R^2^	0.002			0.03		
F	1.16			9.22		

Mediate variables: sleep disorders, moderated variables: HbA1c, independent variables: SSBs, dependent variables: BMI. The model was uncontrolled covariates.

**Table 12:** Bootstrapped conditional direct and indirect effects.

			BMI		
Direct effect			Effect	SE	(LL,UL)
	Predictor	BMI			
	Moderator (HbA1c)	Low	-1.63	0.49	-2.59,-0.67
		Medium	-1.43	0.35	-2.13,-0.74
		High	-1.24	0.43	-2.08,-0.39
Indirect effect			Effect	SE	(LL,UL)
	Predictor	BMI			
	Mediator (sleep disorders)	Low	5.83	-0.03	-0.14,0.01
		Medium	7.58	-0.02	-0.11,0.06
		High	9.33	0.06	-0.07,0.23

## Discussion

### The correlation between SSBs and obesity

The overall prevalence of obesity and overweight was 71.5% among 1765 patients with T2DM, which was similar to a previous study (18). This shows that diabetes combined with obesity is a common phenomenon, suggesting that we should pay more attention to the harm of this phenomenon. Here, the frequency of no SSB intake was more than 50%, and the average scores of sleep disorders and HbA1c values were 7.05 ± 1.28 and 7.58 ± 1.75%, respectively. An association between SSBs, sleep disorders, and diabesity was also observed. We also explored sex differences in HRFs, and we found that some HRFs, such as HbA1c, FBG and TG, showed higher levels in male participants, whereas TC level was higher in female participants.

Obesity is a complex condition caused by various factors, including genetics, epigenetics, diet and food environment, physical activity, metabolism, psychosocial influences, and environmental factors (19, 20). Although diabetes has been recognized for a long time, only a few studies have been conducted to study the factors associated with it and the potential moderator mechanisms. Therefore, we explored the correlation between SSBs, sleep patterns, and WC and BMI. Our sensitivity analysis also explored the relationship between dietary behaviors (including SSBs, juice, and tuber crops), sleep disorders, WC, and BMI. Moreover, sleep patterns and SSBs consumption also have been identified as appropriate targets for public health interventions since they are strongly correlated with obesity and provide only biological rhythm disorder and “empty” calories with little nutritional value. Additionally, unhealthy lifestyle choices, characterized by an unbalanced diet and inadequate sleep, contribute to metabolic changes that can result in the onset of non-communicable diseases (NCDs) (21, 22).

Previous studies discovered a weak correlation between SSBs and T2DM, which is fairly consistent in epidemiological studies according to ecological data, with BMI and WC having little effect on SSBs and T2DM. However, better BMI had a more pronounced effect on SSB and T2DM, which corresponds with our results. There is a positive correlation between SSBs consumption and diabesity (including BMI and WC level). Additionally, BMI is an important influencing factor, reflecting the complex interaction between SSB and caloric intake in obesity and T2DM progression (23). This trend also provides a theoretical basis for our results which shows that SSBs correlate with obesity and T2DM. Furthermore, numerous studies have shown that in patients with T2DM and obesity, more intensive dietary energy restriction and very low-calorie diets significantly reduce HbA1c and fasting glucose levels promoting sustained diabetes remission (24, 25) and lipid management for at least 2 years (26). Therefore, considering lifestyle and glucose management, we examined the associations and possible mechanisms leading to diabetes occurrence. Here, we found a three-way interaction effect between HbA1c, sleep disorders, and SSBs on BMI and WC. Additionally, we manipulated the process of HbA1c, sleep disorders, SSBs, TC, and TG in our sensitivity analysis, demonstrating that diabetes may be caused by the interaction of multiple factors. The differences in sex were similar to those shown in other studies, and increasing evidence suggests that sex affect the pathophysiology, incidence, prevalence, symptoms and signs, disease course and response to treatment of many diseases (27). Moreover, the T2DM prevalence differs between men and women as, the prevalence of diabetes is slightly higher in women than in men (28). The reason difference between HRFs and diabesity and it is now obvious that many aspects of energy balance and glucose metabolism are distributed differently and regulated by different factors in men and women and influence their susceptibility to type 2 diabetes (27).

### The moderation relationship

The correlation between individual risk factors has been mentioned in the previous discussion; whereas whether there is a correlation between these risk factors is also worth exploring. SSBs are correlated with T2DM and CVD risk, partly because they can induce weight gain and insulin resistance (29). However, the independent effect may also result from the high number of carbohydrates that can be absorbed quickly, such as any form of sugar food, the primary sweetener incorporated in SSBs. Furthermore, consumption of SSBs can lead to rapid and significant increases in blood glucose and insulin concentrations, which, combined with frequent heavy consumption, leads to a high dietary glycemic load (GL) (30). Moreover, previous studies have found that patients with high SSBs had an odd ratio (OR) (without adjusting for obesity) of 2.56 for T2DM. The OR was slightly changed by adjusting for BMI, WC, or total body fat; however, the OR was significantly reduced by adjusting for estimated body fat. These indicators had similar impacts on the relationship between SSB and T2DM, with HbA1c unknown > 6.5% and > 48 mmol/mol in patients with T2DM (23). Lifestyle modification in individuals with diabetes refers to dietary restrictions, such as SSBs intake, with the benefit of SSBs restriction accomplished through BMI control (12).

We also explored sleep disorder-related diabetes since it is well established that sleep/wakefulness regulation is related to the circadian rhythms, which is the body’s internal clock that controls metabolic processes. The master clock in the suprachiasmatic nucleus (SCN) of the hypothalamus regulates the sleep drive that causes regular daily sleep patterns. Furthermore, sleep duration and disorders are rough markers of circadian rhythm stages. However, since light is the most potent stimulus that alters circadian rhythms, sleep terminates on light exposure, and its timing affects circadian rhythms. Therefore, sleep and the circadian rhythms are intertwined. Specifically, abnormal sleep may affect the pathway to T2DM associated with obesity, and sleep deprivation leads to elevated nocturnal catecholamine levels in the laboratory (31, 32). Considerable evidence reveals that short sleep duration may be a risk factor for obesity due to obesity-promoting hormone secretion (33). Chaput et al. also found a relationship between shorter sleep duration and higher intake of regular soft drinks (34). In our moderation analysis study, we also examined that the sex difference between SSBs, sleep disorders and diabesity, according to previous study (27), they raised that biological sex as a determinant of energy balance and body composition. Attention to gender differences plays an important role in the subsequent diagnosis and treatment of diabetes, and the complex mechanisms responsible for the metabolic regulation of sex dimorphism need to be further described. The contribution of sex chromosomes to dimorphic gene expression in metabolic tissues provides interesting new insights (35).

Additionally, there is accumulating evidence that inadequate sleep (i.e., short and/or poor sleep quality) is associated with obesity and other harmful health outcomes, and the major mechanism between insufficient sleep and weight gain results from increased food intake, especially high-energy foods, providing a theoretical basis for our results. Furthermore, the relationship between obesity and metabolic indices and sleep was considered from the standpoint of patients with diabetes. Therefore, screening for habitual sleep patterns in patients with diabetes is critical (20). Morselli et al. concluded that there was a relationship between shorter sleep duration and the incidence of diabetes mellitus and/or obesity and that inadequate sleep could increase food intake. Accordingly, this review also demonstrated that screening for habitual sleep patterns in patients with “diabesity” is crucial (36). Observational studies can provide better insight into the role and relative importance of potential biological pathways that link HRFs to chronic disease by measuring relevant intermediate agents on these pathways and performing formal mediation analysis (37). Therefore, this information will advance our scientific knowledge of the potential causal pathways and underlying mechanisms and could also help identify potential targets for intervention, particularly considering the limitations of conducting long-term randomized controlled trials (38).

Moreover, these risk factors also collectively lead to insulin resistance, hyperinsulinemia, atherogenic dyslipidemia, hypertriglyceridemia, reduced HDL-C, and increased LDL-C levels (39); thus, diabetes forms a subset of metabolic syndrome. Another study found that diet and lifestyle interventions significantly contributed to the clinical management of these conditions (40). PREDIMED-Plus has also extensively gained further insights into lifestyle interventions, including the monitoring of cognitive symptoms (41), sleep quality and patterns (42), and the benefits of promoting and prescribing moderate and moderate-to-vigorous physical activity for an improvement in inflammatory profiles and individuals’ health (40).

## Conclusion

Sleep disorders and SSBs intake are modifiable risk factors for preventing and treating diabetes and metabolic diseases and promoting healthy metabolism. WC is currently available in most health surveys with a slightly better correlation with total body fat and indicating the body fat distribution (41). Here, WC and BMI were employed as outcome variables to observe the influencing factors that may lead to diabetes combined with obesity (diabesity) since studies have shown that WC and BMI can be indicators of diabetes severity prognosis (33, 40). Furthermore, early diagnosis of the effects of diabetes on the cardiovascular system would facilitate the optimal application of effective therapies to prevent further complications (40). Therefore, additional studies are warranted to explore further the predictive value of these HRFs for clinical outcomes and, ultimately, as potential surrogate HRFs in a large and growing cohort of patients with diabetes worldwide.

### Strength and limitations

The study’s strengths include the following: many foreign studies have reported HRFs and diabetes and obesity and its complications; therefore, this study applied SSBs and sleep pattern correlations to investigate metabolic indices, which provides a solid theoretical foundation. Additionally, this study used a multicenter and multilevel design and several participants.

This review had some limitations. First, imprecision and recall bias associated with self-report measures imply that their ability to assess total sleep patterns accurately and SSBs is limited. Moreover, since the design of SSBs is significantly broad, there is a certain recall bias in this part of the dataset. Second, as a cross-sectional study, it was difficult to observe a causal relationship between HRFs, sleep patterns, SSBs, BMI, and WC. Finally, this study only investigated the results of four cities. Therefore, the representativeness of this sample is unclear and would require a follow-up survey to be performed among samples from different regions and cultures across the country.

## Data availability statement

The raw data supporting the conclusions of this article will be made available by the authors, without undue reservation.

## Ethics statement

The studies involving human participants were reviewed and approved by the First Affiliated Hospital of Anhui Medical University Ethics Committee. The patients/participants provided their written informed consent to participate in this study.

## Author contributions

QZ and the China National Diabetic Chronic Complications Study Group constructed the study design. QZ recruited the participants. YZ and CL were involved in statistical analysis. QZ were responsible for the critical revision of the manuscript. YZ and QZ edited and revised the manuscript. YZ, CL YW and YLZ prepared and drafted the manuscript. All the authors who contributed to the manuscript gave their approval for its submission. The work presented here has not been published previously and is not being considered for publication elsewhere. The author (s) read and approved the final manuscript. All authors have read and approved the manuscript.
