# Supplementary material for: The relationship between sugar-sweetened beverages, sleep disorders, and diabesity
Source: Front Endocrinol (Lausanne). 2023 Jan 9;13:1041977. doi: 10.3389/fendo.2022.1041977 (PMC9869278; doi:10.3389/fendo.2022.1041977)
Supplement: Supplementary file 1 [file DataSheet_1.doc]

**Supplement table**

| Table S1 The sex difference in health risk factors (t test) | | | | | | |
| --- | --- | --- | --- | --- | --- | --- |
| Variables | Male |  |  | Female |  |  |
|  | Mean | se |  | Mean | se | P |
| Physical activity (moderate) | 0.27 | 0.70 |  | 0.23 | 0.62 | 0.14 |
| Sedentary behavior | 3.88 | 2.62 |  | 3.64 | 2.60 | 0.058 |
| Walking | 1.45 | 0.50 |  | 1.43 | 0.50 | 0.49 |
| Sleep disorders | 6.85 | 1.21 |  | 7.25 | 1.31 | **0.00** |
| Sleep duration | 6.93 | 1.66 |  | 6.86 | 1.83 | 0.38 |
| HbA1c | 7.68 | 1.76 |  | 7.48 | 1.73 | **0.02** |
| FBG | 9.63 | 3.30 |  | 9.24 | 3.21 | **0.01** |
| TG | 2.48 | 3.35 |  | 2.17 | 1.67 | **0.012** |
| TC | 5.11 | 1.27 |  | 5.25 | 1.11 | **0.013** |

| Table S2 The sex difference in health risk factors (Chi-square test) | | | |
| --- | --- | --- | --- |
| Variables | Male | Female | *χ^2^* value |
| Alcohol drinking |  |  | 340.86** |
| Yes | 465(34.2) | 108(18.8) |  |
| No | 408(34.2) | 784(65.8) |  |
| Smoking |  |  | 625.82** |
| Yes | 498(95.4) | 24(4.6) |  |
| No | 375(30.2) | 868(69.8) |  |
| SSBs |  |  | 0.37 |
| Yes | 59(52.2) | 54(47.8) |  |
| No | 814(49.3) | 838(50.7) |  |
| Sleep (dichotomy) |  |  | 1.30 |
| ≥7h | 537(50.6) | 525(49.4) |  |
| ＜7h | 336(47.8) | 367(52.2) |  |

| Table S3 The multilevel linear regression between dependent health risk factors and WC | | | | | | | | | | | |
| --- | --- | --- | --- | --- | --- | --- | --- | --- | --- | --- | --- |
| Variables | Male | | | | |  | Female | | | | |
|  | R^2^ | t | F | P | β (95%CI) |  | R^2^ | t | F | P | β (95%CI) |
| Physical activity (moderate) | 0.13 | -0.014 | 2.40 | ＞0.05 | -0.007(-0.94,0.92) |  | 0.16 | -1.22 | 3.63 | ＞0.05 | -0.66(-1.72,0.40) |
| Sedentary behavior | 0.15 | 2.02 | 3.08 | **＜0.05** | 0.26(0.007,0.51) |  | 0.15 | 0.17 | 3.38 | ＞0.05 | 0.022(-0.23,0.28) |
| Walking | 0.13 | 1.14 | 2.62 | ＞0.05 | 0.76(-0.55,2.07) |  | 0.15 | 0.80 | 3.49 | ＞0.05 | 0.54(-0.79,1.87) |
| Sleep (dichotomy) | 0.13 | 0.92 | 2.54 | ＞0.05 | 0.62(-0.70,1.93) |  | 0.16 | 1.48 | 3.75 | ＞0.05 | 1.0(-0.33,2.33) |
| Alcohol drinking | 0.14 | 1.38 | 2.72 | ＞0.05 | 0.92(-0.39,2.22) |  | 0.15 | 0.22 | 3.39 | ＞0.05 | 0.22(-1.78,2.21) |
| Smoking | 0.13 | -0.37 | 2.42 | ＞0.05 | -0.25(-1.55,1.05) |  | 0.16 | 1.34 | 3.69 | ＞0.05 | 2.75(-1.27,6.77) |
| SSBs | 0.13 | 0.71 | 2.48 | ＞0.05 | 0.93(-1.63,3.49) |  | 0.19 | 3.69 | 5.70 | **＜0.01** | 5.09(2.38,7.79) |
| Sleep disorders | 0.024 | 2.54 | 3.49 | **＜0.01** | 0.69(0.16.1.23) |  | 0.037 | 3.72 | 5.73 | **＜0.01** | 0.96(0.45,1.46) |
| Sleep duration | 0.13 | -0.27 | 2.41 | ＞0.05 | -0.05(-0.44,0.33) |  | 0.16 | -1.17 | 3.61 | ＞0.05 | -0.21(-0.57,0.15) |
| HbA1c | 0.13 | -0.10 | 2.41 | ＞0.05 | -0.02(-0.38,0.35) |  | 0.18 | 0.10 | 2.85 | **＜0.01** | 0.54(0.17,0.92) |
| FBG | 0.13 | -1.15 | 2.63 | ＞0.05 | -0.12(-0.31,0.08) |  | 0.15 | 1.27 | 3.58 | ＞0.05 | 0.13(-0.07,0.33) |
| TG | 0.19 | 4.25 | 5.54 | **＜0.01** | 0.42(0.22,0.61) |  | 0.21 | 4.51 | 6.77 | **＜0.01** | 0.89(0.50,1.27) |
| TC | 0.13 | 0.94 | 2.62 | ＞0.05 | 0.24(-0.27,0.75) |  | 0.15 | -0.65 | 3.38 | ＞0.05 | -0.20(-0.78,0.39) |
| Controlled for educational level, total annual household income, marital status, ethnic,and age. | | | | | | | | | | | |

| Table S4 The multilevel linear regression between dependent health risk factors and BMI | | | | | | | | | | | |
| --- | --- | --- | --- | --- | --- | --- | --- | --- | --- | --- | --- |
| Variables | Male | | | | |  | Female | | | | |
|  | R^2^ | t | F | P | β (95%CI) |  | R^2^ | t | F | P | β (95%CI) |
| Physical activity (moderate) | 0.14 | 0.29 | 3.03 | ＞0.05 | 0.05(-0.30,0.40) |  | 0.11 | -0.97 | 1.93 | ＞0.05 | -0.20(-0.60,0.20) |
| Sedentary behavior | 0.16 | 2.19 | 3.83 | **＜0.05** | 0.10(0.01,0.20) |  | 0.11 | 0.76 | 1.87 | ＞0.05 | 0.04(-0.06,0.13) |
| Walking | 0.15 | 0.89 | 3.16 | ＞0.05 | 0.22(-0.27,0.71) |  | 0.12 | 1.20 | 2.01 | ＞0.05 | 0.31(-0.20,0.81) |
| Sleep (dichotomy) | 0.16 | -2.23 | 3.87 | ＞0.05 | -0.35(-0.65,-0.04) |  | 0.11 | -0.28 | 1.79 | ＞0.05 | -0.04(-0.35,0.26) |
| Alcohol drinking | 0.15 | 0.83 | 3.14 | ＞0.05 | 0.20(-0.28,0.69) |  | 0.11 | 0.60 | 1.83 | ＞0.05 | 0.23(-0.52,0.98) |
| Smoking | 0.14 | -0.58 | 3.08 | ＞0.05 | -0.14(-0.62,0.34) |  | 0.11 | -0.35 | 1.79 | ＞0.05 | -0.27(-1.78,1.25) |
| SSBs | 0.15 | 1.16 | 3.25 | ＞0.05 | 0.56(-0.39,1.51) |  | 0.18 | 4.13 | 4.65 | **＜0.01** | 2.14(1.12,3.16) |
| Sleep disorders | 0.17 | 2.46 | 4.05 | **＜0.05** | 0.25(0.05,0.45) |  | 0.17 | 3.95 | 4.40 | **＜0.01** | 0.38(0.19,0.57) |
| Sleep duration | 0.15 | 0.69 | 3.10 | ＞0.05 | 0.05(-0.09,0.19) |  | 0.11 | -0.85 | 1.89 | ＞0.05 | -0.06(-0.19,0.08) |
| HbA1c | 0.16 | -1.74 | 3.54 | ＞0.05 | -0.12(-0.26,0.02) |  | 0.11 | 0.80 | 1.82 | ＞0.05 | 0.06(-0.08,0.20) |
| FBG | 0.17 | -2.63 | 4.20 | **＜0.05** | -0.1(-0.17,-0.03) |  | 0.11 | -0.29 | 1.73 | ＞0.05 | -0.01(-0.09,0.07) |
| TG | 0.23 | 5.33 | 7.93 | **＜0.01** | 0.19(0.12,0.26) |  | 0.16 | 3.49 | 3.77 | **＜0.01** | 0.26(0.11,0.41) |
| TC | 0.15 | 0.51 | 3.15 | ＞0.05 | 0.05(-0.14,0.24) |  | 0.12 | -1.51 | 2.09 | ＞0.05 | -0.17(-0.39,0.05) |
| Controlled for educational level, total annual household income, marital status, ethnic,and age. | | | | | | | | | | | |
