# Supplementary material for: The relationship between sugar-sweetened beverages, sleep disorders, and diabesity
Source: Front Endocrinol (Lausanne). 2023 Jan 9;13:1041977. doi: 10.3389/fendo.2022.1041977 (PMC9869278; doi:10.3389/fendo.2022.1041977)
Supplement: Supplementary file 2 [file DataSheet_2.doc]

**Methods**

***Sampling setting***

Phase I: 123 out of 298 monitoring sites of chronic diseases and their risk factors in China were selected as the survey sites. According to the provinces (autonomous regions, municipalities directly under the Central Government), the survey points are divided into two levels: district and county. Two districts and two counties were randomly selected from each province. (1) Xizang and Qinghai are difficult to investigate, so one district and one county are selected in these two provinces, and one more county is selected in Sichuan, Henan and Shandong provinces with large population. (2) Tianjin, Shanghai and Beijing consider that there are few county-level units, and adjust county-level units to regional sampling units. Finally, (31×4-1) = 123 monitoring points were selected from 298 monitoring points at the district and county level as the investigation points.

Phase II: Chronic diseases and their risk factors in China in 2013 were selected from each selected survey point, 4 streets (towns) measured.

Phase III: In 2013, chronic diseases and their risk factors in China were monitored for diabetic patients and basic males. The diabetes patients managed by the general health service were stratified by gender and age first, and then by gender and age ratio 120 cases were randomly selected for investigation.

***Data collection and management***

The data collection of each survey content of each survey point shall be the survey software uniformly compiled and issued by the national project working group. After the end of the daily survey, the tablet network needs to be uploaded to the central server. Each provincial project working group shall regularly check the quality of data input from each survey point and timely feedback any problems. After the provincial data report, the national project working group will clean up the data in the investigation point, finally summarize the national data for analysis, and feedback the cleaned database to the provincial project working group, and the provincial project working group will feedback the data to each investigation point.

***Definitions of major variables***

Dyslipidemia: Total cholesterol (TC) > 5.18 mmol/L and/or (TG) triglyceride TG > 1.70 mmol/L and/or high-density lipoprotein cholesterol (HDL-C) <1.04 mmol/L and/or low density lipoprotein cholesterol (LDL-C) > 3.37 mmol/L [1]. (Having a history of dyslipidemia and Hypertension disease in the past one year, and/or currently receiving treatment with lipid-lowering medications was regarded as dyslipidemia and Hypertension in this study). Hypertension: systolic pressure > 140 mmHg or diastolic pressure > 90 mmHg [2]. BMI: For the Chinese population, obesity is defined as a BMI of ≥ 28 kg/m^2^, and overweight as a BMI of 24–27.9 kg/m^2^ [3]. Central obesity: waist circumference (WC) > 80 cm for females and WC > 85 cm for males [4]. Age groups: according to the criteria of age classification by WHO reported in 2012, age range is divided into three groups: young (18–44 years), middle (44–59 years) and old (≥ 60 years) [3].

**Reference**

[1] He H, Yu YQ, Li Y, Kou CG, Li B, Tao YC, Zhen Q, Wang C, Kanu JS, Huang XF, Han M, Liu YW. Dyslipidemia awareness, treatment, control and influence factors among adults in the Jilin province in China: a cross-sectional study. Lipids in health and disease. 2014;13:122. doi: 10.1186/1476-511x-13-122.

[2] Yang G, Ma Y, Wang S, Su Y, Rao W, Fu Y, Yu Y, Kou C. Prevalence and Correlates of Prehypertension and Hypertension among Adults in Northeastern China: A Cross-Sectional Study. Int J Environ Res Public Health. 2015;13:82. doi: 10.3390/ijerph13010082.

[3] Wang R, Zhang P, Gao C, Li Z, Lv X, Song Y, Yu Y, Li B. Prevalence of overweight and obesity and some associated factors among adult residents of northeast China: a cross-sectional study. BMJ Open. 2016;6:e010828. doi: 10.1136/bmjopen-2015-010828.

[4] Zhang P, Wang R, Gao C, Jiang L, Lv X, Song Y, Li B. Prevalence of Central Obesity among Adults with Normal BMI and Its Association with Metabolic Diseases in Northeast China. PLoS One. 2016;11:e0160402. doi: 10.1371/journal.pone.0160402.

**Results**

***3.3* *The multilevel linear regression between dependent health risk factors, BMI, WC and SSBs***

In Table S5, controlled for covariates, some health risk factors and BMI,WC were positively correlated with SSBs. Smoking, BMI, WC and TG were correlated with SSBs.

| Table S5 The multilevel linear regression between dependent health risk behaviors and SSBs | | | | | |
| --- | --- | --- | --- | --- | --- |
| Variables | SSBs | | | | |
|  | R2 | t | F | P | β |
| Physical activity (moderate) | 0.008 | -0.855 | 2.028 | ＞0.05 | -0.008(-0.025,0.01) |
| Sedentary behavior | 0.008 | 0.45 | 1.952 | ＞0.05 | 0.001(-0.003,0.006) |
| Walking | 0.008 | 0.902 | 2.04 | ＞0.05 | 0.011(-0.013,0.034) |
| Sleep (dichotomy) | 0.009 | 1.406 | 2.207 | ＞0.05 | 0.017(-0.007,0.04) |
| Alcohol drinking | 0.01 | 1.849 | 2.415 | ＞0.05 | 0.026(-0.002,0.053) |
| Smoking | 0.015 | 3.533 | 3.720 | **＜0.01** | 0.056(0.025,0.087) |
| BMI | 0.015 | 3.701 | 3.895 | **＜0.01** | 0.006(0.003,0.009) |
| WC | 0.013 | 3.037 | 3.521 | **＜0.01** | 0.002(0.001,0.003) |
| Sleep disorders | 0.008 | 0.779 | 2.01 | ＞0.05 | 0.004(-0.006,0.013) |
| Sleep duration | 0.009 | -1.462 | 2.231 | ＞0.05 | -0.005(-0.011,0.002) |
| HbA1c | 0.009 | 1.716 | 2.368 | ＞0.05 | 0.006(-0.001,0.012) |
| FBG | 0.008 | 1.708 | 2.112 | ＞0.05 | 0.002(-0.002,0.005) |
| TG | 0.01 | 1.981 | 2.537 | **＜0.05** | 0.004(0.000,0.009) |
| TC | 0.008 | 0.227 | 1.979 | ＞0.05 | 0.001(-0.008,0.011) |
| Controlled for educational level, marital status, ethnic, gender and age. *P＜0.05，**P＜0.01 | | | | | |

***3.6 The moderation analysis between SSBs, FBG, HbA1c and WC, BMI***

Moderation analyses were performed with educational level, total annual household income, marital status, ethnic, gender and age as the control variables, the results are presented in Table S6. First, SSBs significantly possible associates of the severity of WC (β = -22.85), HbA1c was not associated with WC (β = -1.63), FBG also not significantly possible associates of the severity of WC (β = -2.09). Secondly, there were significant effect between HbA1c and SSBs on WC (β = 2.80), there are also three-way interaction effect between HbA1c, FBG and SSBs on WC (β = -0.32).

| Table S6 The moderation analysis between SSBs, FBG, HbA1c and waist circumference | | | | | | |
| --- | --- | --- | --- | --- | --- | --- |
| Variables | Waist circumference | | | | | |
|  | coeff | se | t | P | LLCI | ULCI |
| HbA1c | -1.6364 | 1.43 | -1.15 | ＞0.05 | -4.4315 | 1.1587 |
| SSBs | -22.8531 | 10.16 | -2.25 | **0.0246** | -42.7770 | -2.9292 |
| Int_1 | 3.1601 | 1.24 | 2.56 | **0.0106** | 0.7364 | 5.5838 |
| FBG | -2.0908 | 1.29 | -1.62 | ＞0.05 | -4.6211 | 0.4394 |
| Int_2 | 2.8046 | 1.16 | 2.41 | **0.0160** | 0.5240 | 5.0851 |
| Int_3 | 0.2163 | 0.12 | 1.73 | ＞0.05 | -.0283 | 0.4610 |
| Int_4 | -0.3164 | 0.11 | -2.91 | **0.0037** | -0.5299 | -0.1029 |
| Int 1: SSB × HbA1c; Int 2: SSB × FBG; Int 3: HbA1c × FBG; Int 4: SSB × HbA1c × FBG | | | | | | |

Moderation analyses were controlled for covariates, the results are presented in Table S7. First, SSBs significantly predicted the severity of BMI (β = -9.10), HbA1c was not associated with BMI (β = -0.77), FBG significantly possible associates of the severity of BMI (β = -0.98). Secondly, there were significant effect between HbA1c and SSBs on BMI (β = 1.21), SSB × FBG on BMI (β = 1.20), HbA1c × FBG on BMI (β = 0.09); there were also three-way interaction effect between HbA1c, FBG and SSBs on BMI (β = -0.13).

| Table S7 The moderation analysis between SSBs, FBG, HbA1c and BMI | | | | | | |
| --- | --- | --- | --- | --- | --- | --- |
| Variables | BMI | | | | | |
|  | coeff | se | t | P | LLCI | ULCI |
| HbA1c | -0.77 | 0.53 | -1.44 | ＞0.05 | -1.80 | 0.27 |
| SSBs | -9.10 | 3.78 | -2.41 | **0.0161** | -16.51 | -1.69 |
| Int_1 | 1.21 | 0.46 | 2.64 | **0.0085** | 0.31 | 2.11 |
| FBG | -.98 | 0.48 | -2.04 | **0.0411** | -1.92 | -0.04 |
| Int_2 | 1.20 | 0.43 | 2.78 | **0.0055** | 0.35 | 2.05 |
| Int_3 | 0.09 | 0.05 | 2.05 | **0.0409** | 0.004 | 0.19 |
| Int_4 | -0.13 | 0.04 | -3.21 | **0.0014** | -0.20 | -0.05 |
| Int 1: SSB × HbA1c; Int 2: SSB × FBG; Int 3: HbA1c × FBG; Int 4: SSB × HbA1c × FBG | | | | | | |

***3.7 The moderation analysis between*** ***HbA1c, SSBs, sleep disorders and TC, TG (sensitivity analyses)***

Moderation analyses were performed with educational level, marital status, total annual household income, ethnic, gender and age as the control variables. The results are presented in Table S8. First, SSBs significantly possible associates of the severity of TC (β = 0.90), sleep disorders was also associated with TC (β=0.556), SSBs × sleep disorders significantly predicted the severity of TC (β = -0.53).

| Table S8 The moderation analysis between SSBs, sleep disorders and TC | | | | | | |
| --- | --- | --- | --- | --- | --- | --- |
| Variables | TC | | | | | |
|  | coeff | se | t | P | LLCI | ULCI |
| Sleep disorders | 0.56 | 0.27 | 2.09 | **0.0367** | 0.34 | 1.08 |
| SSBs (dichotomy) | 0.90 | 0.42 | 2.14 | **0.0321** | 0.77 | 1.72 |
| Int_1 | -0.53 | 0.24 | -2.19 | **0.0288** | -1.01 | -0.06 |
| Int 1: SSB(dichotomy) × sleep disorders | | | | | | |

Moderation analyses were performed with educational level, marital status, total annual household income, ethnic, gender and age as the control variables. The results are presented in Table S9. First, SSBs significantly predicted the severity of TC (β = 0.0032), sleep disorders was not associated with TC (β = -0.0005), SSBs × sleep disorders significantly predicted the severity of TC (β = -0.0014).

| Table S9 The moderation analysis between SSBs, sleep disorders and TC | | | | | | |
| --- | --- | --- | --- | --- | --- | --- |
| Variables | TC | | | | | |
|  | coeff | se | t | P | LLCI | ULCI |
| Sleep disorders | -0.0005 | 0.05 | -0.01 | ＞0.05 | -0.089 | 0.088 |
| SSBs (continuity) | 0.0032 | 0.002 | 2.06 | **0.0393** | 0.0002 | 0.006 |
| Int_1 | -0.0014 | 0.001 | -2.04 | **0.0412** | -0.003 | -0.0001 |
| Int 1: SSB (continuity) × sleep disorders | | | | | | |

Moderation analyses were performed with educational level, marital status, total annual household income, ethnic, gender and age as the control variables. The results are presented in Table S10. SSBs significantly predicted the severity of TG (β = 1.05), sleep disorders was also associated with TG (β = 2.44), SSBs × sleep disorders significantly predicted the severity of TG (β = -0.86).

| Table S10 The moderation analysis between SSBs, sleep disorders and TG | | | | | | |
| --- | --- | --- | --- | --- | --- | --- |
| Variables | TG | | | | | |
|  | coeff | se | t | P | LLCI | ULCI |
| Sleep disorders | 1.0546 | 0.41 | 2.57 | **0.0102** | 0.25 | 1.86 |
| SSBs (continuity) | 2.4352 | 0.89 | 2.73 | **0.0063** | 0.69 | 4.18 |
| Int_1 | -0.8643 | 0.37 | -2.31 | **0.0209** | -1.60 | -0.13 |
| Int 1: SSB × sleep disorders | | | | | | |

Moderation analyses were performed with educational level, marital status, total annual household income, ethnic, gender and age as the control variables. The results are presented in Table S11. First, SSBs not significantly predicted the level of TC (β = -4.62), sleep disorders was also not associated with TC (β = -1.05), HbA1c significantly predicted the level of TC (β = -1.59). Secondly, there were also do not have significant effect between SSBs and sleep disorders on TC (β = 0.50), SSB × HbA1c on TC (β = 0.82), HbA1c × sleep disorders on TC (β = 0.19); there are three-way interaction effect between HbA1c, sleep disorders and SSBs on TC (β = -0.09).

| Table S11 The moderation analysis between SSBs, FBG, HbA1c and TC | | | | | | |
| --- | --- | --- | --- | --- | --- | --- |
| Variables | TC | | | | | |
|  | coeff | se | t | P | LLCI | ULCI |
| Sleep disorders | -1.0507 | 0.68 | -1.51 | ＞0.05 | -2.39 | 0.29 |
| SSBs | -4.6166 | 2.88 | -1.60 | ＞0.05 | -10.26 | 1.03 |
| Int_1 | 0.5005 | 0.35 | 1.42 | ＞0.05 | -0.19 | 1.19 |
| HbA1c | -1.5889 | 0.71 | -2.23 | **0.0258** | -2.98 | -0.19 |
| Int_2 | 0.8238 | 0.37 | 2.24 | **0.0255** | 0.10 | 1.55 |
| Int_3 | 0.1909 | 0.086 | 2.22 | **0.0264** | 0.022 | 0.6 |
| Int_4 | -0.0926 | 0.045 | -2.079 | **0.0378** | -0.18 | -0.005 |
| Int 1: SSBs × sleep disorders; Int 2: SSBs × HbA1c; Int 3: HbA1c × sleep disorders;  Int 4: SSBs × sleep disorders × HbA1c | | | | | | |

Moderation analyses were performed with educational level, marital status, total annual household income, ethnic, gender and age as the control variables. The results are presented in Table S12. First, SSBs significantly predicted the level of TG (β = -12.50), sleep disorders was not associated with TG (β = -2.82), HbA1c significantly predicted the level of TG (β = -3.30). Secondly, there were also have significant effect between sleep disorders and SSBs on TG (β = 1.53), SSB × HbA1c on TG (β = 1.92), HbA1c × sleep disorders on TG (β = 0.44); there are three-way interaction effect between HbA1c, sleep disorders and SSBs on TG (β = -0.24).

| Table S12 The moderation analysis between SSBs, FBG, HbA1c and TG | | | | | | |
| --- | --- | --- | --- | --- | --- | --- |
| Variables | TG | | | | | |
|  | coeff | se | t | P | LLCI | ULCI |
| Sleep disorders | -2.8168 | 1.49 | -1.89 | ＞0.05 | -5.74 | 0.11 |
| SSBs | -12.4978 | 6.31 | -1.98 | **0.0477** | -24.87 | -0.12 |
| Int_1 | 1.5322 | 0.77 | 1.98 | **0.0476** | 0.017 | 3.05 |
| HbA1c | -3.2955 | 1.56 | -2.11 | **0.0347** | -6.35 | -0.24 |
| Int_2 | 1.9212 | 0.81 | 2.38 | **0.0174** | 0.341 | 3.50 |
| Int_3 | 0.4399 | 0.19 | 2.34 | **0.0195** | 0.07 | 0.81 |
| Int_4 | -0.2418 | 0.10 | -2.52 | **0.0199** | -0.43 | -0.05 |
| Int 1: SSBs × sleep disorders; Int 2: SSBs × HbA1c; Int 3: HbA1c × sleep disorders;  Int 4: SSBs × sleep disorders × HbA1c | | | | | | |
